# Postpartum Transient Hypervagotonic Sinus Node Dysfunction Leading to Sinus Bradycardia: A Case Report

**DOI:** 10.7759/cureus.9186

**Published:** 2020-07-14

**Authors:** Roshan Acharya, Rajesh Shrestha

**Affiliations:** 1 Internal Medicine, Cape Fear Valley Hospital, Fayetteville, USA; 2 Internal Medicine, Campbell University School of Osteopathic Medicine, Fayetteville, USA; 3 Internal Medicine, Rhode Island Hospital, Providence, USA

**Keywords:** sinus node dysfunction, postpartum pain, increased vagal tone, hypervagotonic, sinus bradycardia

## Abstract

Sinus bradycardia is common in children and adults, especially during sleep. The heart rate can drop below 30 beats per minute. Up to 35% of healthy individuals below 25 years of age, trained athletes, and those with a rare form of the familial syndrome with potassium/sodium hyperpolarization-activated cyclic nucleotide-gated channel 4 (HCN4) mutation may have asymptomatic sinus bradycardia without any heart diseases. The increased vagal tone has been associated with profound bradycardia in various pathophysiologic settings including pain. Herein, we report the first case of a young Caucasian female with transient symptomatic bradycardia due to postpartum hypervagotonic sinus node dysfunction (SND).

## Introduction

Sinus bradycardia is defined as the heart rate < 60 beats per minute. It can be due to various medical conditions like increased intracranial pressure, myxedema, hypothermia, and vasovagal response [1]. It can occur in normal individuals as well as in various pathophysiological settings where the vagal tone is increased, like during sleep [2]. Furthermore, it can be a normal physiological phenomenon especially in young adults between the age group of 20 to 30 years [3]. Any emotional stressors and painful stimuli can directly activate the medullary cardiovascular centers via the hypothalamus and increase vagal tone, which in return inhibits the firing of the sinoauricular node leading to bradycardia. This mechanism is a well-known explanation for the bradycardia and syncope during a vasovagal episode [4]. Fortunately, sinus bradycardia itself is not associated with increased mortality, without any sinoatrial dysfunction [1].

## Case presentation

**Figure 1:**
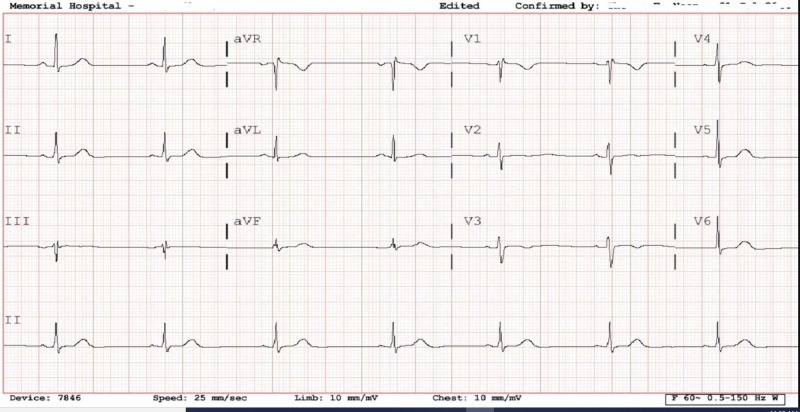
EKG showing sinus bradycardia

A 28-year-old Caucasian female without any significant past medical history presented to the emergency room with complaints of three-day history of palpitation and exertional dyspnea. She underwent a lower segment cesarean section under spinal anesthesia without any complications 10 days ago and was discharged on opiates for pain relief. However, her pain recurred after stopping opiates. She denied any fever, discharge from the incision site, or abnormal lochia. She used to smoke a half pack of cigarettes but quit before pregnancy. Family history was unremarkable and, she had no allergies. Vital signs at the time of admission were as follows; heart rate of 31 beats/minute, blood pressure of 150/63 mmHg, respiratory rate was 16/min and she was afebrile. Physical examination was positive for mild tenderness in the lower abdomen. Otherwise, the rest of the physical examination was normal. Laboratory investigation revealed normal hemogram (hemoglobin of 11.6 g/dL, hematocrit of 33.1%, platelets 217,000 per mm^3^), normal electrolytes including magnesium and calcium, normal thyroid, liver, and kidney function tests. Electrocardiogram (EKG) revealed sinus bradycardia without any atrioventricular block and ST-segment and T wave changes (Figure 1). Cardiac enzymes and chest radiograph were normal. She was admitted to the telemetry floor with the preliminary diagnosis of postpartum symptomatic sinus bradycardia secondary to increased vagal tone likely due to postpartum pain following cesarean section. During the hospital stay, an echocardiogram revealed normal ejection fraction without any signs of cardiomyopathy. She was subsequently discharged home. A 30-day outpatient Holter monitor was unremarkable without any atrioventricular blocks and pauses. At a six-week follow-up, bradycardia and clinical symptoms had resolved.

## Discussion

Park et al. demonstrated that hypervagotonic sinus node dysfunction (SND) can be asymptomatic but can also present with dizziness, syncope, dyspnea, weakness, or fatigue [5]. The study demonstrated EKG findings were variable and the most common presentation was sinus bradycardia, followed by sinus pause, sinoatrial block, and tachycardia-bradycardia. Hypervagotonic SND was diagnosed if abnormal electrophysiological properties of the sinus node returned to normal after the administration of atropine [5]. Nevertheless, there are not enough pieces of evidence, available to clarify the clinical characteristics and response to drug therapy. And however, non-hypervagotonic SND is one of the major indications for pacemaker implantation [6].

The parasympathetic innervations of the heart are supplied by the vagus nerve through the right and left branches. The right vagus nerve supplies sinuatrial node (SAN) and left supplies atrio-venticular node (AVN) [7]. When there is increased vagal tone, it can predispose to bradyarrhythmias and heart blocks [2]. The vagus nerve control heart rate directly by influencing pacemaker current (If) in SAN in the right atrium [8]. The cholinergic nerve terminal in SAN uses acetylcholine as a neurotransmitter which is responsible for the negative chronotropic effect on the heart by inhibiting the hyperpolarization-activated pacemaker current (If) [5].

The SND is not only caused by intrinsic abnormalities but also by various extrinsic factors like drugs, infections, and autonomic dysfunctions like increased vagal tone [3,5]. The enhanced parasympathetic tone can be physiological like in sleep, or drugs that mimic parasympathetic stimulation like lithium, or due to pathological conditions. The pathological conditions mainly include parasympathetic nerve-rich organs like gastrointestinal tract, genitourinary, oropharynx, etc [5]. The hypervagotonic SND can be seen in highly conditioned athletes. Though hypervagotonia is an uncommon cause of syncope, it should be considered in well-trained athletes experiencing syncope associated with significant bradycardia in the absence of structural heart disease [9]. Obstructive sleep apnea (OSA), on the other hand, can cause severe sinus bradycardia (heart rate < 30 beats per minute) during apneic periods [10]. Rarely, vagus nerve stimulation (VNS) therapy, a treatment modality for refractory epilepsy, can cause late-onset bradycardia and syncope. The cause remains unclear but the anatomic variation of the vagus nerve or the change in sensitivity of afferent nerve endings and medulla oblongata due to chronic VNS has been attributed [11]. Methylergonovine, an ergot alkaloid, used to control postpartum hemorrhage has also been reported to cause late-onset sinus bradycardia [12]. However, our patient didn’t have any of the abovementioned risk factors to explain the phenomena.

The treatment of the hypervagotonic SND is different from the intrinsic SND. Hypervagotonic SND is suspected by its transient course and its association with vagally mediated symptoms like nausea and vomiting. However, it can be confirmed if atropine reverses the electrophysiologic abnormalities of SAN [5]. The hypervagotonic SND can be treated safely without any pacemaker. A study demonstrated the relative efficacy of theophylline to treat SND. In this study, the patients were treated with oral theophylline and followed for a long period (43+/-28 months) of time. It caused remission of the symptoms even after discontinuation in about 70% of the patients and only one patient (3.1%) needed permanent pacemaker [5]. Hypervagotonic sinus bradycardia associated with other conditions gradually improves as the predisposing factors improve. For example in OSA, sinus bradycardia tends to improve as the OSA improves with treatment, and in highly trained athletes, sinus bradycardia is treated with the cessation of the exercise. However, pacemaker insertion is only needed for those athletes who are unwilling to quit exercising [9,10]. Permanent pacemaker insertion is recommended in symptomatic patients excluding the correctable external causes of SND [13]. Our patient didn’t have abnormalities in EKG and during Holter monitoring other than sinus bradycardia, which improved during follow-up period. It is not always possible to diagnose SND from standard EKG or Holter monitoring [14]. To our knowledge, this is the first report of transient hypervagotonic SND in a postpartum woman following the cesarean section, which improved without any intervention.

## Conclusions

Hypervagotonic SND can be seen in various clinical settings like extreme pain, carotid massage, OSA, vasovagal syncope and in highly trained athletes, where enhanced vagal tone plays the major role. It is transient in nature and the subtle EKG findings are extremely difficult to identify the condition in normal clinical settings. With the knowledge and understanding of basic pathophysiology of increased vagal tone in different physiological and pathological clinical settings and its effect on heart rate, we can avoid patients from unnecessary investigations and interventions.
